# The Effects of ATIR Blocker on the Severity of COVID-19 in Hypertensive Inpatients and Virulence of SARS-CoV-2 in Hypertensive hACE2 Transgenic Mice

**DOI:** 10.1007/s12265-021-10147-3

**Published:** 2022-01-01

**Authors:** Xiaoliang Jiang, Huadong Li, Yong Liu, Linlin Bao, Lingjun Zhan, Hong Gao, Wei Deng, Jing Xue, Jiangning Liu, Xing Liu, Junli Li, Jie Wang, Shuang Wu, Mingzhe Yan, Wei Luo, Pedro A. Jose, Chuan Qin, Xiuhong Yang, Dingyu Zhang, Zhiwei Yang

**Affiliations:** 1 Key Laboratory of Human Disease Comparative Medicine Chinese Ministry of Health, Beijing Key Laboratory for Animal Models of Emerging and Remerging Infectious Diseases Institute of Laboratory Animal Science, Chinese Academy of Medical Sciences and Comparative Medicine Center, Peking Union Medical College, 5 Pan Jia Yuan Nan Li, Chaoyang District, Beijing, 100021 People’s Republic of China; 2 Jinyintan Hospital, Wuhan, China, Dongxihu District, Wuhan, 430023 People’s Republic of China; 3 Department of Orthopedics, Peking Union Medical College Hospital, Chinese Academy of Medical Sciences and Peking Union Medical College, Beijing, 100730 People’s Republic of China; 4 Department of Clinical Laboratory, General Hospital of Tianjin Medical University, No.154, Anshan Road, Heping District, Tianjin, 300052 People’s Republic of China; 5 Division of Renal Diseases and Hypertension, Department of Medicine, and Department of Pharmacology/Physiology, The George Washington University School of Medicine and Health Sciences, Washington, DC 20052 USA; 6 Department of Physiology, School of Basic Medical Sciences, North China University of Science and Technology, 57 Jianshe South Rd, Tangshan City, Hebei 063000 People’s Republic of China

**Keywords:** Coronavirus disease 2019, Hypertension, AT1 receptor blocker, Angiotensin converting enzyme 2

## Abstract

Angiotensin-converting enzyme 2 (ACE2) is required for the cellular entry of the severe acute respiratory syndrome coronavirus 2. ACE2, via the Ang-(1-7)-Mas-R axis, is part of the antihypertensive and cardioprotective effects of the renin-angiotensin system. We studied hospitalized COVID-19 patients with hypertension and hypertensive human(h) ACE2 transgenic mice to determine the outcome of COVID-19 with or without AT1 receptor (AT1R) blocker treatment. The severity of the illness and the levels of serum cardiac biomarkers (CK, CK-BM, cTnI), as well as the inflammation markers (IL-1, IL-6, CRP), were lesser in hypertensive COVID-19 patients treated with AT1R blockers than those treated with other antihypertensive drugs. Hypertensive hACE2 transgenic mice, pretreated with AT1R blocker, had increased ACE2 expression and SARS-CoV-2 in the kidney and heart, 1 day post-infection. We conclude that those hypertensive patients treated with AT1R blocker may be at higher risk for SARS-CoV-2 infection. However, AT1R blockers had no effect on the severity of the illness but instead may have protected COVID-19 patients from heart injury, via the ACE2-angiotensin1-7-Mas receptor axis.

**Supplementary Information:**

The online version contains supplementary material available at 10.1007/s12265-021-10147-3.

## Introduction

The coronavirus disease 2019 (COVID-19) pandemic caused by severe acute respiratory syndrome coronavirus 2 (SARS-CoV-2) is a major public health problem. Cardiovascular diseases, including hypertension and diabetes, are the most common comorbidities that are associated with severe illness and high case-fatality rates (CFR) in COVID-19 [1, 2]. SARS-CoV-2 is a newly identified coronavirus with high genome similarity (89%) and identity (73%) of its receptor-binding domain (RBD) and similar ternary structure to SARS-CoV [3–5]. Molecular modeling revealed that SARS-CoV-2 RBD has a stronger interaction with angiotensin-converting enzyme 2 (ACE2), as cell entry receptor, than SARS-CoV [5]. ACE2 is a homologue of ACE but has physiological actions that oppose ACE [6]. ACE cleaves angiotensin I to generate angiotensin II which binds and stimulates the AT1 receptor (AT1R) to increase blood pressure, in part, via arterial constriction and renal sodium retention [7]. By contrast, ACE2 catalyzes the conversion of angiotensin (Ang) II into Ang1-7 which leads to vasodilatory, antiproliferative, and anti-inflammatory effects via the Mas receptor. Thus, ACE2 counterbalances the effects of Ang II and exhibits antihypertensive and cardioprotective effects in humans and several rodent models [8].

AT1R blockers, such as losartan and olmesartan, are widely used in the therapy of hypertension. Urinary ACE2 levels are increased in hypertensive patients treated with the AT1R blocker (olmesartan), but not in patients treated with other AT1R blockers, such as losartan, candesartan, valsartan, and telminsartan [9]. However, losartan and other AT1R blockers have been reported to increase ACE2 expression in the kidney of spontaneously hypertensive rat and heart of mice or rat with cardiac hypertrophy and dysfunction caused by transverse aortic constriction or coronary artery ligation [10, 11]. It has been suggested that angiotensin receptor 1 (AT1R) blockers may be used to reduce the cardiac damage in SARS-CoV-2 infection [12]. Retrospective studies found that hospitalized hypertensive patients with SARS-CoV-2 infection treated with ACE inhibitors or AT1R blockers have a lower risk of all-cause mortality than those treated with other drugs [13]. A prospective cohort study also found that ACE inhibitors and AT1R blockers are associated with a reduced progression of COVID-19 and do not increase the risk of intensive care [14]. However, due to ACE2 is critically important in the cellular entry of SARS and SARS-CoV-2, it is possible that hypertensive COVID-19 patients treated with drugs that increase ACE2, e.g., ACE inhibitors and AT1R blockers, are at a higher risk for the development and severity SARS-CoV-2 infection [15]. Indeed, hypertensive patients taking ACE inhibitors or AT1R blockers may have higher all-cause mortalities than those taking other drugs [16]. It has been suggested that ACE2-mediated inhibition of the AT1R pathway, which is beneficial in the treatment of hypertension, may also increase the susceptibility to pulmonary and cardiovascular disease in patients with COVID-19 [17]. Human (h) ACE2 transgenic mice, intranasally inoculated with SARS-CoV-2, develop severe pulmonary pathology with viral replication in the lungs and intestines, at 1-day post infection (dpi) and severe pulmonary pathology 5 dpi. There is no viral load in other organs [18]. The disease is more severe in aged mice [19], similar to that seen in humans [20]. The intranasal inoculation of SARS-CoV-2 in human (h) ACE2 transgenic mice results in 100% mortality within a few days [21]. However, the effect of AT1R blockers in hypertensive hACE2 transgenic mice, induced by angiotensin II and infected with SARS-CoV-2, has not been studied. Therefore, we studied hospitalized COVID-19 hypertensive patients and hypertensive hACE2 transgenic mice to determine whether AT1R blockers protect or promote the development of COVID-19 [17].

## Methods

See the supplementary data for details.

## Results

### AT1R Blockers May Protect Hypertensive COVID-19 Patients Against Inflammation and Heart Injury

One-hundred forty-six hospitalized COVID-19 patients with hypertension were investigated, including 48 patients who took AT1R blockers for hypertension treatment (AT1RB group) and 98 patients who took other antihypertensive medications (Non-AT1RB group) before admission. After admission, all of them stopped taking all antihypertensive medications, including AT1R blockers, because of the concern that the latter such medications may worsen the outcome of SARS-CoV-2 infection. The age and sex distribution, and number of days of illness prior to admission, were similar in the two groups (Table 1). However, the severity of the disease, some symptoms, and some physical findings were lesser in the AT1RB than the non-AT1RB group, which could indicate that AT1R blockers may have reduced the severity of SARS-CoV-2 infection in these hypertensive patients, which was no dose-dependent effect.

**Table 1:** Demographics and clinical characteristics on admission of hypertensive COVID-19 inpatients treated with AT1R blockers or non-AT1R blockers

Variables	AT1R blockers (n = 48)	Non-AT1R blockers (n = 98)	P value
Age (years)	62.5 (11.55)	64.6 (9.83)	0.2472
Sex			0.7246
Male, n (%) Female, n (%)	23 (47.9) 25 (52.1)	50 (51.0) 48 (49.0)	
BMI (kg/m^2^)	26.2 ± 3.1	27.5 ± 4.3	0.265
Blood pressure			
Systolic (mm Hg) Diastolic (mm Hg)	137.4 ± 17.7 84.1 ± 11.5	134.8 ± 17.4 82.7 ± 10.8	0.2754 0.1889
Clinical severity			0.0293*
Mild, n (%) Severe, n (%) Critical, n (%)	30 (62.5) 15 (31.3) 3 (6.3)	43 (43.9) 42 (42.9) 13 (13.3)	
Medical history, n (%)			
Nephropathy Cerebral infarction Heart disease Diabetes Hyperlipidemia	1 (2.1) 3 (6.2) 5 (10.4) 8 (16.6) 14 (29.1)	2 (2.0) 5 (5.1) 12 (12.2) 20 (20.4) 21 (21.4)	0.6439 0.5941 0.5783 0.4132 0.2291
Outcome			
Recovery, n (%) Disability, n (%) Death, n (%)	34 (70.8) 2 (4.2) 12 (25.0)	65 (66.3) 2 (2.0) 31 (31.6)	0.5241
Days of illness prior to admission, n (%)	11.0 (4.73)	12.2 (5.15)	0.1717
Symptoms and physical findings			
Cough, n (%) Fever, n (%) Runny nose, n (%) Sputum production, n (%) Myalgia/fatigue, n (%) Headache, n (%) Diarrhea, n (%) Respiratory rate (breaths/min) Oxygen saturation (%) Heart rate (beats/min)	33 (68.8) 41 (85.4) 0 (0) 19 (9.6) 34 (70.8) 4 (8.3) 2 (4.2) 20.0 ± 1.98 95.0 ± 4.81 97 ± 5.76	76 (78.4) 78 (79.6) 3 (3.1) 22 (22.4) 52 (53.1) 3 (3.1) 8 (8.2) 21.5 ± 1.06 92.5 ± 3.15 93 ± 9.76	0.2079 0.3944 0.5510 0.0305* 0.0403* 0.3230 0.5827 0.0077* 0.1531 0.2823

Continuous variables with normal distribution are expressed as mean ( ± SD) and compared using Student’s *t*-test; continuous variables with non-normal distribution are expressed as median (IQR) and compared using Wilcoxon rank sum test; categorical variables are expressed as number (%) and compared using χ^2^ test, Fisher’s exact test, or Wilcoxon rank sum test. *AT1RB* patients on AT1R blockers, *No-AT1RB* patients on antihypertensive treatment other than AT1R blockers, *BMI* body mass index.

*P<0.05 vs. AT1R blockers

**Fig. 1:**
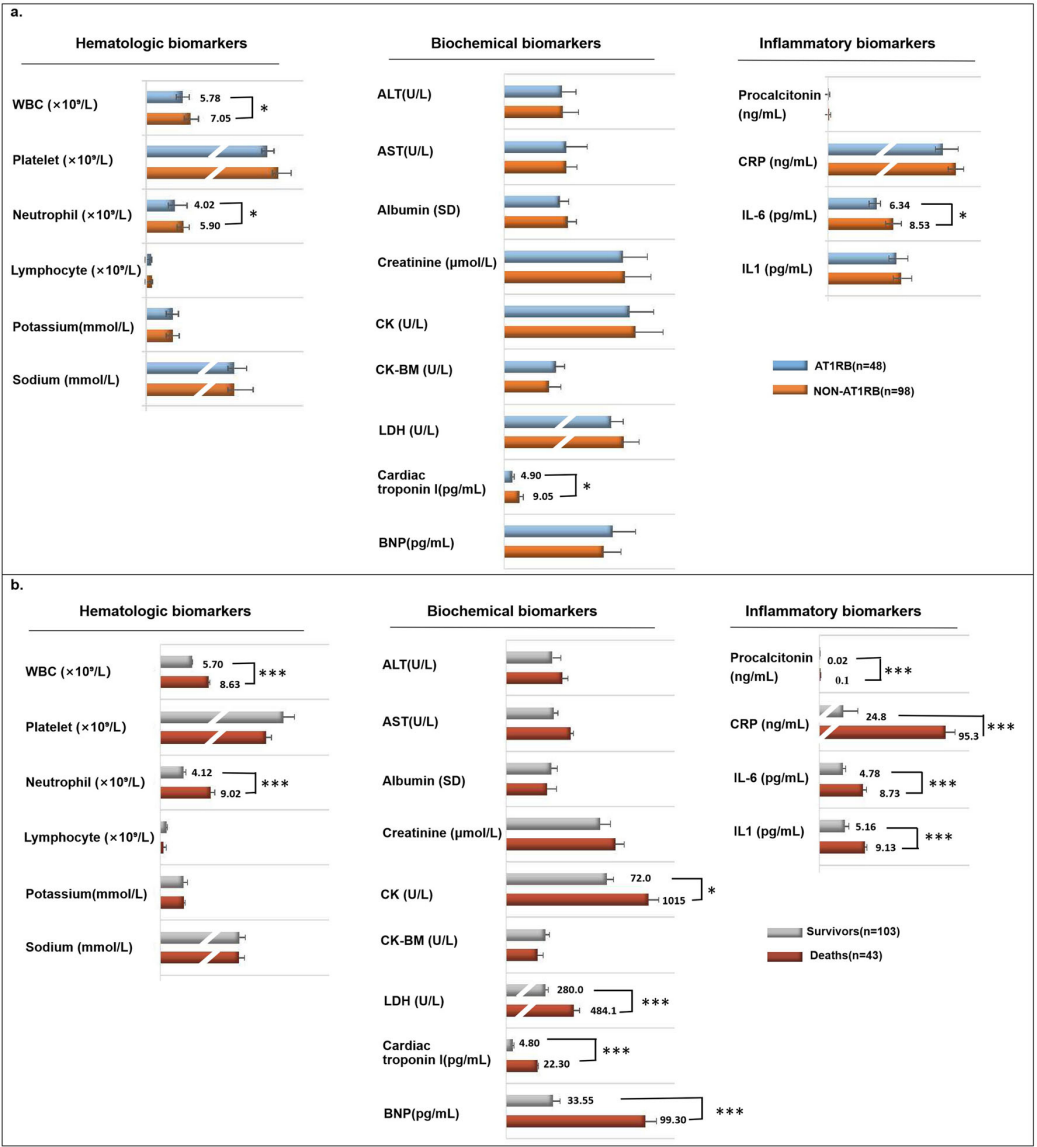
Laboratory test results in hypertensive COVID-19 patients with or without AT1R blocker treatment (**a**) and surviving or non-surviving hypertensive COVID-19 patients (**b**). Continuous variables with normal distribution were expressed as mean (SD) and compared using Student’s *t*-test; categorical variables were expressed as number (%) and compared using χ^2^ test or Fisher’s exact test. AT1RB = patients on AT1R blockers; Non-AT1RB = patents on antihypertensive treatment other than AT1R blockers. *P<0.01 vs. AT1RB group; ***P<0.0001 vs. survivor group

We then compared the laboratory results on the admission of the two groups. The white blood cell and neutrophil counts, cardiac troponin levels, and interleukin-6(IL-6) levels were lesser in the AT1RB group than those in the non-AT1RB group (Fig. 1a). Therefore, we suggest that AT1R blockade may decrease the inflammation in hypertensive COVID-19 patients.

### Cardiac Function-Related Tests on Admission Are Strongly Associated with the Outcome in COVID-19 Inpatients with Hypertension

In order to investigate the causes of death in all COVID-19 patients with hypertension, we analyzed the results of the laboratory tests on admission in survivors (103 patients) and non-survivors (43 patients) (Fig. 1b). We found that the admission serum levels of most of the cardiac function-related tests, including serum creatinine kinase (CK), lactate dehydrogenase (LDH), cardiac troponin, and NT-proB-type natriuretic peptide (BNP), were higher in non-surviving than surviving hypertensive COVID-19 patients. The white blood cell and neutrophil counts and serum levels of procalcitonin, C-reactive protein (CRP), IL-6, and IL-1 were also higher on admission in non-surviving than surviving patients. By contrast, platelet and lymphocyte counts were higher in the surviving than non-surviving group. Therefore, we presume that cardiac dysfunction and inflammation may be the main causes of death in COVID-19 patients with hypertension (Fig. 1b).

### Hypertensive hACE2 Mice Have an Increased Risk for SARS-CoV-2 Infection

**Fig. 2:**
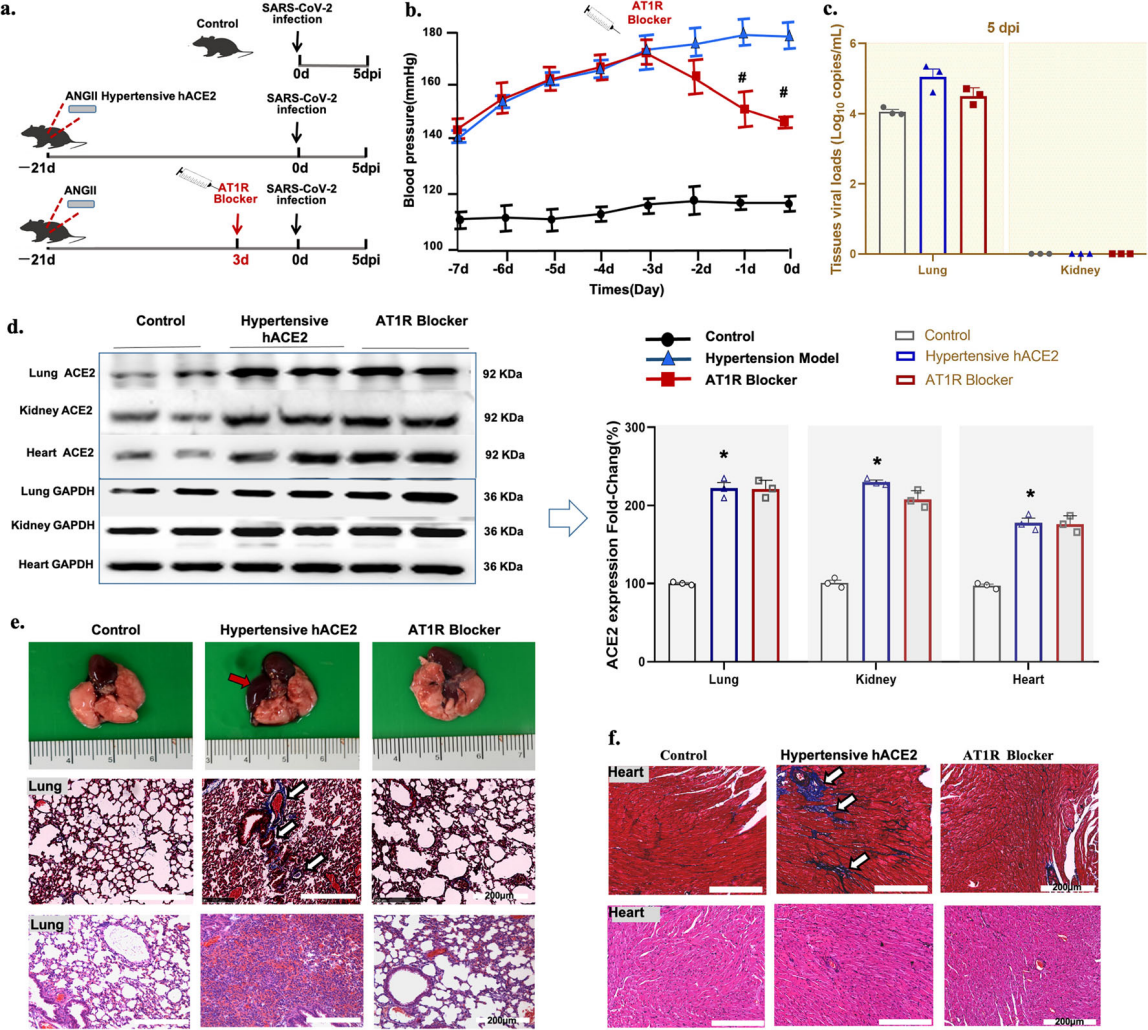
Virus replication and representative histopathology in SARS-CoV-2-infected hACE2 transgenic mice with hypertension induced by Ang II. **a** Protocol for SARS-CoV-2-infection in vivo. **b** Systolic blood pressure (SBP) was measured by tail-cuff plethysmography in conscious mouse, three times at 8:30 AM. **c** The SARS-CoV-2 load at 5 dpi. The SARS-CoV-2 loads in the lung and kidney of the mice were quantified by qRT-PCR. **d**Angiotensin-converting enzyme 2 (ACE2) expression quantified by western blot and analyzed by ImageJ (n=3/group). Data are presented as mean ± SD. **e** Morphology image (top), Masson-stained(middle) and H&E-stained(bottom) sections of the lung (scale bar is 200 μm) and heart (**f**) from control (normotensive hACE2 transgenic mice) and hypertensive hACE2 transgenic mice without and with AT1R blocker treatment (5 dpi). *P<0.01 vs. control (untreated normotensive hACE2 transgenic mice), ^#^P<0.05 vs. untreated hypertensive hACE2 transgenic mice, Wilcoxon rank. Data are presented as mean ± SD

To assess whether high blood pressure and organ dysfunction contribute to the increased risk for SARS-CoV-2 infection, hACE2 transgenic mice were induced hypertensive by high salt feeding (1% NaCl) and subcutaneous infusion of angiotensin II (Ang II, 500 ng/mg/day), via osmotic minipumps, for 3 weeks before SARS-CoV-2 infection. The lungs, kidneys, and hearts were harvested at 5 dpi (Fig. 2a). Control hACE2 transgenic mice (black line) were normotensive group. By contrast, Ang II-infusion in hACE2 transgenic mice (blue line) caused a progressive increase in systolic blood pressure from an initial value of 105 ± 4.1 to 178±8.3 mm Hg, 3 weeks (0d)(Fig. 2b). At 5 dpi, no viral load was detected in the kidney, but the viral load was higher in the lungs of hACE2 hypertensive transgenic mice than in the normotensive hACE transgenic mice (control)(Fig. 2c). Relative to the normotensive hACE transgenic mice (control), the expression of ACE2 was increased in three organs (lung, kidney, and heart) of hypertensive hACE2 mice (Fig. 2d), which may increase the risk for SARS-CoV-2 infection.

### AT1R Blocker Treatment Protects Hypertensive hACE2 Transgenic Mice From SARS-CoV-2-Mediated Organ Injury

To investigate the effect of AT1R blocker on tissue injury post SARS-CoV-2 infection in hypertensive mice, the morphology and histopathology of the lung, heart, and kidney were evaluated at 5 dpi. There was pulmonary edema with bleeding in hypertensive hACE2 transgenic mice, which was not detected in the AT1R blocker-treated hypertensive hACE2 transgenic mice (Fig. 2e). The lung morphology was similar to the non-SARS-CoV-2-infected normotensive hACE2 transgenic group (control). Increased lung and myocardial fibrosis were observed by Masson’s trichrome staining in the hypertensive hACE2 transgenic mice that was improved by AT1R blocker treatment. At 5 dpi, the lung pathology progressed into severe interstitial pneumonia, with thickened alveolar septa in the untreated hypertensive hACE2 transgenic mice with SARS-CoV-2 infection (Fig. 2e). There was also some interstitial pneumonia in the control normotensive hACE2 transgenic mice, which is consistent with our previous study [22, 23]. MMP2, MMP9, and FN-1 expressions were upregulated in the lung and heart (also kidney) which induced increased fibrosis and necrosis in the hypertensive hACE2 transgenic mice (Fig. S1). Thus, AT1R blocker treatment did not aggravate the histopathological injury, but instead protected the organs from damage caused by SARS-CoV-2 in hypertensive hACE2 transgenic mice.

### AT1R Blocker Treatment May Increase the Risk of SARS-CoV-2 Infection in the Heart and Kidney at the Early Period of Infection

In order to study the risk of SARS-CoV-2 infection that may be caused by AT1R blockers, hypertensive hACE2 transgenic mice were pretreated with the AT1R blocker, losartan (6.4ng/kg/day), for 3 days, before the SARS-Cov-2 virus infection as above (Fig. 2a). AT1R blocker treatment (red line) attenuated the increase in BP (145 ± 2.9 vs 178 ± 8.3 mm Hg, 0d) induced by Ang II infusion (Fig. 2b). As shown above, the viral load at 5 dpi was higher in the lungs of the hypertensive hACE2 transgenic mice than control normotensive hACE2 transgenic mice; there was no effect of AT1R blocker on the increase in the viral load (Fig. 2c). However, there was no viral load in the kidney and heart at 5 dpi, regardless of treatment (Fig. 2c).

We then investigated the ACE2 expression and viral replication at 1, 3, and 5 dpi in AT1R blocker-treated and AT1R blocker-untreated (non-Ang II-infused) normotensive hACE2 transgenic mice (Fig. S2a). Western blotting showed that ACE2 protein was increased in the lung, kidney, and heart of the AT1R blocker-pretreated group at 1 and 3 dpi, compared with the normotensive hACE2 transgenic mice. However, these differences were no longer observed at 5 dpi (Fig. S2b). The viral load was similar in the lung of untreated and AT1R blocker-treated normotensive (non-Ang II-infused) hACE2 transgenic mice at 1, 3, and 5 dpi (Fig. S2c). However, a relatively high viral load was observed in the kidney and heart of AT1R blocker-pretreated normotensive mice at 1 dpi. No viral load was detected in the kidney and heart at 3 dpi and 5 dpi (Fig. S2c) with or without AT1R blocker pretreatment in the normotensive hACE2 transgenic mice. These results suggest that AT1R blockade may increase the risk of SARS-CoV-2 infection in the heart and kidney at the early period of infection, even in the absence of hypertension.

In normotensive hACE2 transgenic mice with SARS-CoV-2 infection, there were no differences in histopathological changes in the lung, heart, and kidney at 1, 3, and 5 dpi between the AT1R blocker-untreated and pretreated group (Figs. S3 and S4).

### AT1R Blocker Treatment Decreases the Inflammatory Reaction in Hypertensive hACE2 Transgenic Mice with SARS-CoV-2 Infection

**Fig. 3:**
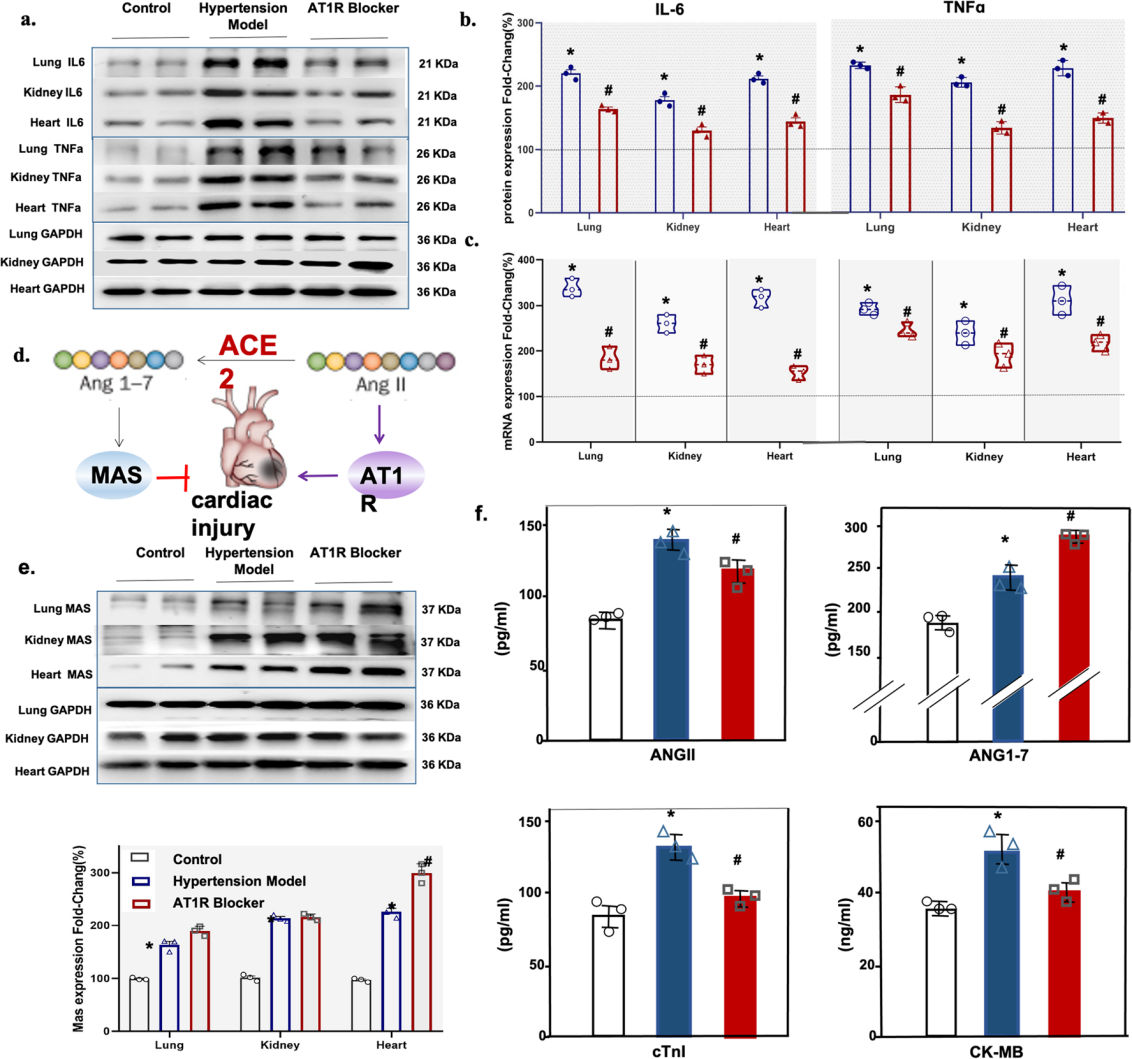
Inflammation and cardiac function markers in SARS-CoV-2-infected normotensive and hypertensive hACE2 transgenic mice with or without AT1R blocker treatment. **a**, **b** Western blots of inflammation markers, IL-6 and TNFα, analyzed by ImageJ. **c** mRNA expression of inflammation markers quantified by qRT-PCR in control (normotensive hACE2 transgenic mice) and hypertensive hACE2 transgenic mice without and with AT1R blocker treatment. **d** Ang II/Ang1-7 imbalance as a key player in cardiac injury. **e** Western blots of cardiac MasR and GAPDH analyzed by ImageJ; **f** serum levels of Ang II, Ang 1-7, cardiac troponin I (cTnI), and creatine kinase-MB(CK-MB) in control normotensive hACE transgenic mice and hypertensive hACE2 transgenic mice without or with AT1R blocker treatment (n=3/group, *P<0.01 vs. control, #P<0.05 vs. untreated hypertensive hACE2 transgenic mice), Wilcoxon rank. Immunoblots (**a** and **e**) are representatives of three independent experiments.

The protein expressions of inflammatory markers (IL-6 and TNFα) were higher in the lung, kidney, and heart, of hypertensive hACE2 transgenic mice than normotensive hACE2 transgenic mice during SARS-CoV-2 infection. The increased expression of inflammatory markers in hypertensive hACE2 transgenic mice was downregulated by AT1R blocker treatment (Fig. 3a and b). The mRNA expressions of two inflammatory factors (IL-6 and TNFα) were also decreased in the lung, kidney, and heart of AT1R blocker-treated hypertensive hACE2 transgenic mice (Fig. 3c). These results indicate that AT1R blocker treatment decreases the inflammatory reaction in hypertensive hACE2 transgenic mice with SARS-CoV-2 infection, which may justify its use in hypertensive patients infected with SARS-CoV-2.

### AT1R Blocker Treatment Protects Hypertensive hACE2 Transgenic Mice from Cardiac Injury by Ameliorating Ang II/Ang1-7 Imbalance

We have found that cardiac dysfunction may be the main cause of death in COVID-19 patients with hypertension [24]. Epidemiological studies also indicated that COVID-19 is more severe in patients with hypertension, coronary heart disease, and diabetes [25]. ACE2-processed Ang-(1-7)-Mas-R axis leads to antihypertensive and cardioprotective effects (Fig. 3d). MasR, the Ang 1-7 receptor, was upregulated in the lung, kidney, and heart of hypertensive hACE transgenic mice with SARS-CoV-2 infection. The expression of MasR in the heart was further increased in hypertensive hACE transgenic mice treated with AT1R blocker; AT1R blocker treatment did not change MasR expression in the lung and kidney (Fig. 3e). Liu et al reported that Ang II levels in COVID-19 patients correlated with viral load and organ injury [26]. We also found increased serum Ang II levels in hypertensive hACE2 transgenic mice, which were decreased by AT1R blocker treatment although the levels were still higher than the untreated hypertensive hACE2 transgenic mice (Fig. 3f). Serum Ang 1–7 levels were also increased in the hypertensive hACE2 transgenic mice, but AT1R blocker treatment further increased the serum Ang 1-7 levels, the opposite of that found with Ang II (Fig. 3f).

We also found that the serum levels of cardiac injury markers, such as cardiac troponin I (cTnI) and creatine kinase-myocardial band (CK-MB), were increased in SARS-CoV-2-infected hypertensive hACE2 transgenic mice but normalized after AT1R blocker treatment (Fig. 3f), which is consistent with the results in our patient study (see above). These results indicate that AT1R blocker may protect hypertensive hACE2 mice from cardiac injury by stimulating the Ang-(1-7)-Mas-R axis and inhibiting the Ang II axis.

## Discussion

The SARS-CoV-2 uses ACE2 as a co-receptor to gain entry inside human cells, where ACE2 is highly expressed, such as the lung, heart, intestine, and kidney, among others [3–5]. SARS-CoV-2 could exploit species-specific interferon-driven upregulation of ACE2 to enhance infection [27]. In the current study, we found that SARS-CoV-2 viral load in the heart and kidney of normotensive hACE2 transgenic mice was increased by AT1R blocker treatment at 1 dpi. The highest SARS-CoV-2 viral load was in the lung of normotensive and hypertensive hACE2 transgenic mice, which was not affected by AT1R blocker treatment. However, the SARS-CoV-2 loading did not increase the severity of injury in the lung, heart, and kidney of normotensive hACE2 transgenic mice with AT1R blocker pretreatment (Supplemental Fig. S3). By contrast, in hypertensive hACE2 transgenic mice, the AT1R blocker treatment was protective against tissue injury and inflammation in the lung, kidney, and heart. ACE2 is critical for early tissue tolerance responses to respiratory infection because elevated ACE2 may increase the ability of the host to tolerate tissue damage [28, 29]. However, while the amount of SARS-CoV-2 load has been reported to predict mortality [30], an effect of AT1R blockers on the relationship between viral load and mortality has not been reported.

In the current study, all hypertensive COVID-19 patients in 15 departments of the hospital during the local epidemic period were investigated to study the severity of SARS-CoV-2 infection in hypertensive COVID-19 patients with or without AT1R blocker treatment. It is very difficult to determine if AT1R blockers affect the severity of SARS-CoV-2 infection in human epidemiological investigation because the time of human contact with the virus is unknown, even though one can compare the number of COVID-19 cases, with or without AT1R blocker treatment, in the population in a specific area. Thus, with a human survey, one can only evaluate the effect of AT1 blocker on the outcome or the severity of SARS-CoV-2 infection. All of the hypertensive COVID-19 patients in our study stopped taking AT1R blockers after admission to the hospital. The intake of AT1R blockers, with the average of 11 days from the onset of illness to the hospital admission of hypertensive COVID-19 patients, suggests that AT1R blockers decreased the severity of SARS-CoV-2 infection. Renin-angiotensin system (RAS) inhibitors have been reported to improve the clinical outcome of COVID-19 patients with hypertension [31]. However, other investigators have reported that neither ACE inhibitors nor AT1R blockers affected the risk of mild-to-moderate or severe COVID-19 disease [32, 33]. In our study, the clinical severity and number of symptoms were less in the AT1R blocker-treated than non-AT1R blocker-treated hypertensive COVID-19 patients. There may be also more recovery and less death in COVID-19 hypertensive patients treated with AT1R blockers. The main causes of death in COVID-19 patients were acute kidney injury, multi-organ failure, inflammatory storm, and thrombo-inflammation [34–39]. Therefore, the laboratory tests on admission could be added to the clinical presentation to determine the effect of ACE inhibitors or AT1R blockers on the severity of SARS-CoV-2 infection.

Acute myocarditis, with elevated blood high-sensitivity troponin T and CK-MB levels, was reported to be associated with the severity of COVID-19, without symptoms and signs of interstitial pneumonia [40]. We also found that the lesser symptoms in COVID-19 patients treated with AT1R blockers were associated with lower serum cTnI levels in hypertensive COVID-19 patients treated with AT1R blockers than those not taking them. The serum cTnI and other cardiac biomarkers were higher in non-survivors than survivors. A meta-analysis study reported that cTnI values were higher in patients with severe than those with milder COVID-19 [41]. Cardiac troponins, i.e., cTnI, are the biomarkers of choice in the diagnosis of acute myocardial infarction in patients in the emergency room [42]. In hACE transgenic mice, the serum levels of cardiac injury markers (cTnI and CK-MB) were higher in hypertensive than normotensive hACE2 mice, which were normalized by AT1R blocker treatment. Therefore, AT1R blockers may decrease the severity of myocardial injury in hypertensive COVID-19 patients.

**Scheme 1:**
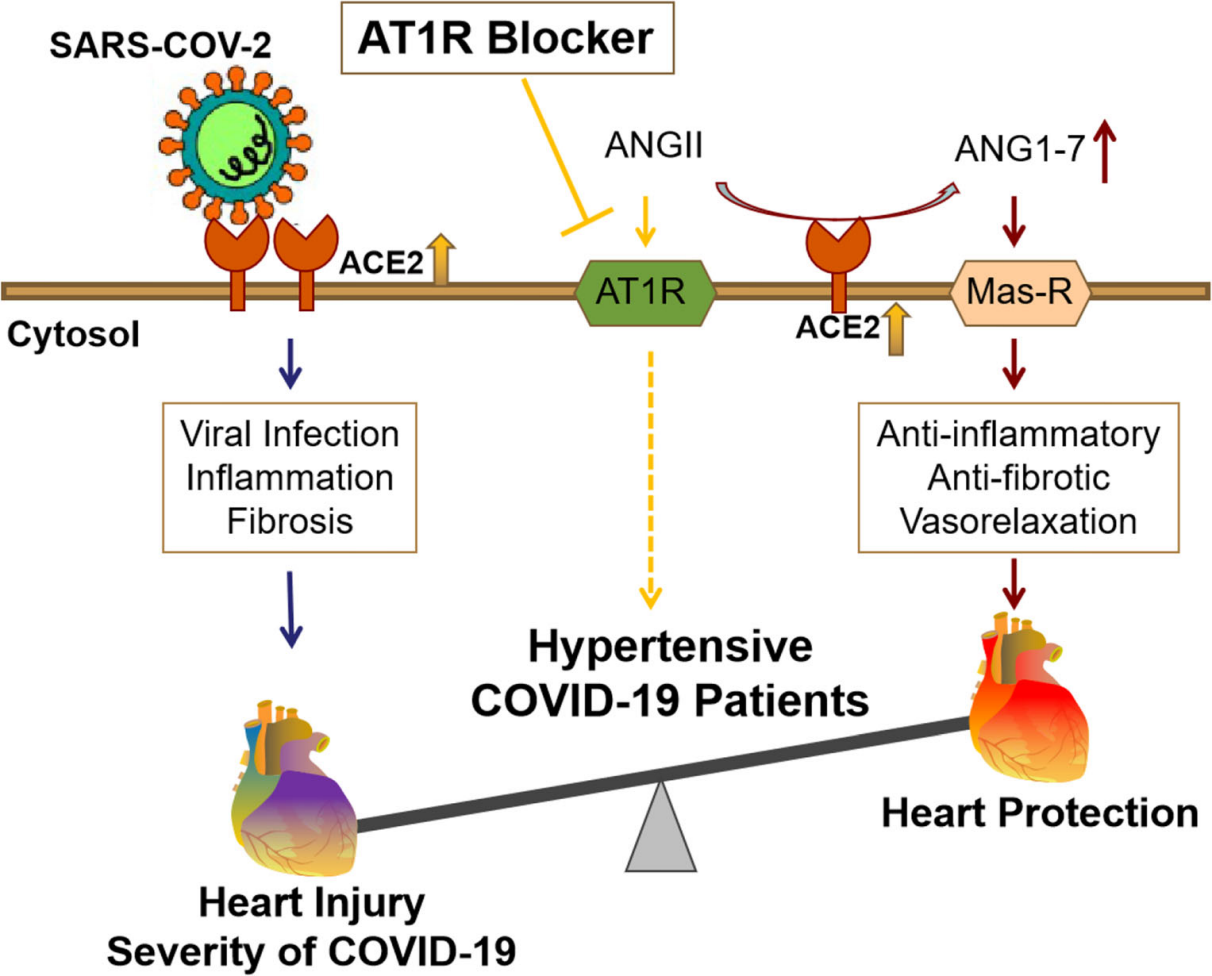
Processing and function scheme of AT1R blocker action in hypertensive COVID-19 patients. Angiotensin II type I receptor (AT1R) blocker increases angiotensin-converting enzyme 2 (ACE2) expression. The increased ACE2 expression may potentiate the risk of SARS-CoV-2 infection, which results in cardiac injury. However, the increase in ACE2 converts more angiotensin (Ang) II to Ang-(1-7), which binds to the Mas receptor (Mas-R) to protect the heart from injury caused by SARS-CoV-2 infection. AT1R blockade also stimulates plasm angiotensin II levels but decreases renal angiotensin II levels [51]

The protective effect of AT1R blockers in hypertensive COVID-19 patients has been reviewed recently [12, 43–45]. As aforementioned, SARS-CoV-2 uses ACE2 to enter the cell [5, 17, 46]. An imbalance in the RAS that favors the Ang II/AT1R axis increases the likelihood of developing severe COVID-19 [46–49]. ACE2 converts Ang II to angiotensin 1-7 (Ang1-7). An increase in ACE2 expression may protect the organs from the virus injury by increasing Ang-(1-7) and decreasing Ang II levels [49, 50]. Ang1-7 binds to the Mas receptor (MasR) that mediates anti-inflammatory, antioxidative, and vasodilatory effects [49, 50]. In hypertensive hACE2 transgenic mice, serum Ang1-7 levels and cardiac MasR expressions were increased, but serum Ang II levels were decreased in AT1R blocker group. We also found that serum Ang-(1-7) levels at 1 dpi were increased in SARS-CoV-2-infected hACE2 transgenic mice with AT1R blocker pretreatment compared with AT1R blocker-untreated mice (data not shown). Therefore, we suggest that AT1R blocker may protect hypertensive COVID-19 patients from heart injury caused by SARS-CoV-2 infection via the ACE2-Ang-(1-7)-Mas-R axis (Scheme 1).

## Perspectives

The clinical severity of SARS-CoV-2 infection was lesser in AT1R blocker-treated than AT1R blocker-untreated hypertensive patients. AT1R blockers were protective against organ injury in hypertensive hACE2 transgenic mice. AT1R blocker treatment was also associated with a decrease in inflammatory markers (TNFα and IL-6). Serum cTnI may be a biomarker that predicts the progression of COVID-19. Although AT1R blockers may increase the risk of SARS-CoV-2 infection in the heart and kidney at the early period of infection, they may be beneficial in the treatment of hypertensive patients with SARS-CoV-2 infection. However, studies in large populations are still needed to evaluate the effect and mechanism of AT1R blockade on the severity and outcome of COVID-19 patients with cardiovascular diseases. More studies are also needed to determine the mechanism of the beneficial effect of AT1R blockers, in general, or specific AT1R blockers, in particular, in hypertensive animal models.

## Limitations

Our study has some limitations. First, this study had to be done in the animal biosafety level 3 (ABSL3) facility, where ultrasound and telemetric measurement of blood pressure are not available. Therefore, we cannot provide cardiac ultrasound data and only tested several serum markers to represent cardiac function. Second, although our study provides additional evidence that ACE2/Ang-1-7/MasR is the protective arm of the RAS, which may countervail the deleterious effects of Ang II in several organs, the mechanisms were not determined. Finally, these studies in Wuhan Chinese may not be generalizable to other racial/ethnic groups.

## Supplementary Information

ESM 1 (DOCX 11651 kb)

## Abbreviations

Ang1-7: Angiotensin 1–7
Ang: Angiotensin
ACE2: Angiotensin-converting enzyme 2
AT1R: AT1 receptor
cTnI: Cardiac troponin I
CFR: Case-fatality rate
COVID-19: Coronavirus disease 2019
CK-MB: Creatine kinase-myocardial band
hs-CRP: High-sensitivity C-reactive protein
IL-6: Interleukin-6
MasR: Mas receptor
RBD: Receptor-binding domains
RAS: Renin-angiotensin system
SARS-CoV-2: Severe acute respiratory syndrome coronavirus 2
